# Supplementary material for: Effects of Shift Work on the Eating Behavior of Police Officers on Patrol
Source: Nutrients. 2020 Apr 4;12(4):999. doi: 10.3390/nu12040999 (PMC7230712; doi:10.3390/nu12040999)
Supplement: Supplementary file 1 [file nutrients-12-00999-s001.pdf]

nutrients

MDPI

Supplementary Tables and Figures

# Effects of shift work on the eating behavior of police officers on patrol

**Anastasi Kosmadopoulos [1], Laura Kervezee [1,2], Philippe Boudreau [1], Fernando Gonzales-Aste [1], Nina Vujovic [3,4], Frank A.J.L. Scheer [3,4,†] and Diane B. Boivin [1,*,†]**

[1] Centre for Study and Treatment of Circadian Rhythms, Douglas Mental Health University Institute, Department of Psychiatry, McGill University, Montreal, Quebec H4H 1R3, Canada
[2] Laboratory for Neurophysiology, Department of Cell and Chemical Biology, Leiden University Medical Center, Leiden 2333 ZC, the Netherlands
[3] Medical Chronobiology Program, Division of Sleep and Circadian Disorders, Departments of Medicine and Neurology, Brigham and Women's Hospital, Boston, Massachusetts 02115, USA
[4] Division of Sleep Medicine, Department of Medicine, Harvard Medical School, Boston, Massachusetts 02115, USA
* Corresponding author
† These authors share senior authorship.

**Table S1.** Sleep, work, and physical activity per day type.

| | Rest Day (n=28) | | Morning Shift (n=21) | | Evening Shift (n=17) | | Night Shift (n=24) | | Effect of Day Type | |
|---|---|---|---|---|---|---|---|---|---|---|
| **Times of Day** | M | SD | M | SD | M | SD | M | SD | *F* (df) [10] | *P* |
| Sleep Onset, pre (hh:mm) [1] | 23:59 [M,E,N] | 0:22 | 22:46 [R,E,N] | 0:20 | 01:27 [R,M,N] | 0:29 | 10:17 [R,M,E] | 0:40 | 150 (3,86) | <0.001 |
| Wake Time, pre (hh:mm) [1] | 08:00 [M,N] | 0:24 | 05:32 [R,E,N] | 0:07 | 08:34 [M,N] | 0:26 | 15:58 [R,M,E] | 0:39 | 146 (3,86) | <0.001 |
| Work Start (hh:mm) | - | - | 06:58 [E,N] | 0:02 | 14:58 [M,N] | 0:02 | 21:53 [M,E] | 0:29 | 3098 (2,59) | <0.001 |
| Work End (hh:mm) | - | - | 16:27 [E,N] | 0:18 | 00:02 [M,N] | 0:10 | 07:51 [M,E] | 0:09 | 5153 (2,59) | <0.001 |
| Sleep Onset, post (hh:mm) [2] | 22:56 [M,E,N] | 0:18 | 22:15 [R,E,N] | 0:15 | 01:11 [R,M,N] | 0:09 | 09:14 [R,M,E] | 0:17 | 560 (3,86) | <0.001 |
| Wake Time, post (hh:mm) [2] | 07:21 [M,E,N] | 0:24 | 06:06 [R,E,N] | 0:20 | 08:22 [R,M,N] | 0:21 | 15:37 [R,M,E] | 0:15 | 258 (3,86) | <0.001 |
| **Durations** | M | SD | M | SD | M | SD | M | SD | *F* (df) [11] | *P* |
| Day Length (h) [3] | 23.38 | 1.40 | 24.63 | 1.32 | 23.78 | 2.15 | 23.64 | 2.63 | 1.8 (3,68) | 0.165 |
| Total Sleep Time (h) [4,9] | 7.90 [N] | 1.00 | 7.44 [N] | 1.64 | 6.86 | 1.52 | 6.27 [R,M] | 1.54 | 7.0 (3,68) | <0.001 |
| Sleep Proportion (%) [5,9] | 33.9 [M,E,N] | 4.4 | 30.0 [R,N] | 5.1 | 28.7 [R] | 5.33 | 26.6 [R,M] | 6.1 | 11.2 (3,64) | <0.001 |
| Work Period (h) | - | - | 9.52 | 1.21 | 9.06 | 0.61 | 10.03 | 1.65 | 2.5 (2,29) | 0.131 |
| **Physical Activity** | M | SD | M | SD | M | SD | M | SD | *F* (df) [11] | *P* |
| Total Activity (cts/min) [6,9] | 292 [N] | 79 | 276 | 55 | 273 | 69 | 251 [R] | 64 | 3.3 (3,58) | 0.027 |
| Activity at Night (cts/min) [7,9] | 63 [M,E,N] | 91 | 142 [R,E,N] | 130 | 229 [R,M] | 58 | 224 [R,M] | 55 | 19.5 (3,63) | <0.001 |
| Activity at Work (cts/min) [8,9] | - | - | 280 [N] | 52 | 255 | 51 | 230 [M] | 52 | 6.5 (2,41) | 0.005 |

[1] Start/end of main sleep period *preceding* shifts/rest days.
[2] Start/end of main sleep period *following* shifts/rest days.
[3] The period starting at the end of the main sleep period preceding shifts/rest days until the end of the main sleep period following.
[4] Total sleep time during naps and the main sleep period following shifts/rest days.
[5] Percentage of the day length spent asleep during naps and the main sleep period following shifts/rest days.
[6] Activity counts per minute from the end of the main sleep preceding shifts/rest days until the start of the following one.
[7] Activity counts per minute of wakefulness between 2300 h–0600 h.
[8] Activity counts per minute from work start to work end.
[9] Evening shift, n=16 due to technical issues.
[10] Circular ANOVA comparing the effect of day type category.
[11] Mixed-effects model ANOVA comparing the effect of day type category, corrected for sex and age.
*P*-values adjusted for multiple comparisons (Benjamini–Hochberg method) by group: Times, Durations, Physical Activity.
[R,M,E,N] = significant pairwise differences (adjusted, $p < 0.05$) between corresponding day types (i.e., Rest, Morning, Evening, Night).

**Table S2.** Counts of meal intakes per day type.

| | Rest Day (n=210) | | Morning Shift (n=133) | | Evening Shift (n=85) | | Night Shift (n=141) | | Effect of Day type [4] | |
|---|---|---|---|---|---|---|---|---|---|---|
| **Meal Intakes** | n | % | n | % | n | % | n | % | $X^2$ (df) | *P* |
| Caloric Ranges | | | | | | | | | 8.58 (12) | 0.739 |
| > 0 to ≤ 5 kcal | 7 | 3.3 | 8 | 6.0 | 3 | 3.5 | 9 | 6.4 | | |
| > 5 to ≤ 100 kcal | 49 | 23.3 | 20 | 15.0 | 14 | 16.5 | 28 | 19.9 | | |
| > 100 to ≤ 500 kcal | 83 | 39.5 | 53 | 39.9 | 37 | 43.5 | 57 | 40.4 | | |
| > 500 to ≤ 1000 kcal | 50 | 23.8 | 38 | 28.6 | 25 | 29.4 | 37 | 26.2 | | |
| > 1000 kcal | 21 | 10.0 | 14 | 10.5 | 6 | 7.1 | 10 | 7.1 | | |
| Meal Types | | | | | | | | | 4.94 (6) | 0.739 |
| Main Meal [1] | 100 | 47.6 | 71 | 53.4 | 44 | 51.8 | 71 | 50.4 | | |
| Snacks | 56 | 26.7 | 37 | 27.8 | 27 | 31.8 | 43 | 30.5 | | |
| Beverage Only | 54 | 25.7 | 25 | 18.8 | 14 | 16.5 | 27 | 19.2 | | |
| At Night [2] | 3 [N] | 1.4 | 6 [N] | 4.5 | 12 [N] | 14.1 | 46 [R,M,E] | 32.6 | 87.84 (3) | <0.001 |
| At Work [3] | - | - | 61 | 45.9 | 34 | 40.0 | 66 | 46.8 | 1.08 (2) | 0.739 |

[1] Calorie intakes classified as breakfast, lunch, or dinner.

[2] Events between 2300 h – 0600 h.

[3] Events between work start and work end.

[4] Chi-square tests of independence, comparing the effect of day type on the proportions of meals of different caloric ranges or meal types, at night or at work.

*P*-values adjusted for multiple comparisons (Benjamini–Hochberg method).

[R,M,E,N] = significant pairwise differences (adjusted, $p < 0.05$) between corresponding day types (i.e., Rest, Morning, Evening, Night).

**Table S3.** Timing of meal intakes and macronutrients per day type.

| | Rest Day (n=28) | | Morning Shift (n=21) | | Evening Shift (n=17) | | Night Shift (n=24) | | Effect of Day type | |
|---|---|---|---|---|---|---|---|---|---|---|
| **Meal Intakes >5 kcal** | M | SD | M | SD | M | SD | M | SD | *F* (df) [6] | *P* |
| First Intake (h) [1] | 1.14 | 0.76 | 1.99 | 1.59 | 1.34 | 0.69 | 1.39 | 2.03 | 1.58 (3,68) | 0.203 |
| Last Intake (h) [2] | 2.55 | 1.11 | 2.08 | 1.17 | 2.97 | 2.52 | 1.94 | 2.36 | 1.58 (3,65) | 0.203 |
| Eating Window (h) [3] | 11.26 [N] | 1.81 | 12.66 | 2.24 | 12.30 | 2.90 | 13.86 [R] | 3.14 | 5.65 (3,63) | 0.005 |
| **Caloric Dispersion** [4] | M | SD | M | SD | M | SD | M | SD | *F* (df) [6] | *P* |
| Total Intake (rho) | 0.563 [N] | 0.131 | 0.459 [N] | 0.115 | 0.502 [N] | 0.179 | 0.356 [R,M,E] | 0.198 | 8.08 (3,84) | <0.001 |
| Carbohydrate (rho) | 0.544 [N] | 0.163 | 0.454 | 0.133 | 0.515 [N] | 0.206 | 0.369 [R,E] | 0.191 | 5.99 (3,66) | 0.002 |
| Fat (rho) | 0.613 [N] | 0.149 | 0.517 | 0.146 | 0.575 [N] | 0.252 | 0.420 [R,E] | 0.237 | 4.65 (3,84) | 0.005 |
| Protein (rho) | 0.568 [N] | 0.11 | 0.513 [N] | 0.154 | 0.573 [N] | 0.203 | 0.374 [R,M,E] | 0.198 | 7.45 (3,84) | <0.001 |
| **Mean Times** [5] | M | SD | M | SD | M | SD | M | SD | *F* (df) [7] | *P* |
| Total Intake (hh:mm) | 15:25 [M] | 0:29 | 14:10 [R] | 0:27 | 14:53 | 0:39 | 23:08 [R,M,E] | 1:14 | 29.27 (3,86) | <0.001 |
| Carbohydrate (hh:mm) | 15:07 [M,N] | 0:31 | 13:26 [R,N] | 0:27 | 14:08 [N] | 0:41 | - [8] | - | 3.99 (2,63) | 0.023 |
| Fat (hh:mm) | 15:16 [N] | 0:35 | 14:11 [N] | 0:36 | 14:53 [N] | 0:45 | 23:06 [R,M,E] | 1:13 | 25.89 (3,86) | <0.001 |
| Protein (hh:mm) | 15:27 [M,N] | 0:27 | 14:16 [R,N] | 0:31 | 15:24 [N] | 0:38 | 22:05 [R,M,E] | 1:01 | 33.17 (3,86) | <0.001 |

[1] Hours after wake time of main sleep period preceding shift/rest day.
[2] Hours before sleep time of main sleep period following shift/rest day.
[3] Duration between first intake event and last intake event.
[4] *Rho*, the mean resultant vector length of circular data, i.e., meal times, weighted by the size of total or macronutrient caloric intake. Smaller values indicate a greater spread or dispersal of calories around the clock.
[5] Circular mean times of caloric intake weighted by the size of total or macronutrient caloric intake.
[6] Mixed-effects model ANOVA comparing the effect of day type category, corrected for sex and age.
[7] Circular ANOVA comparing the effect of day type category.
[8] Excluded because individual carbohydrate mean times were not non-uniformly distributed ($p = 0.097$, Rayleigh test).
*P*-values adjusted for multiple comparisons (Benjamini–Hochberg method) by group: Meal Intakes, Dispersion, Mean Times.
[R,M,E,N] = significant pairwise differences (adjusted, $p < 0.05$) between corresponding day types (i.e., Rest, Morning, Evening, Night).

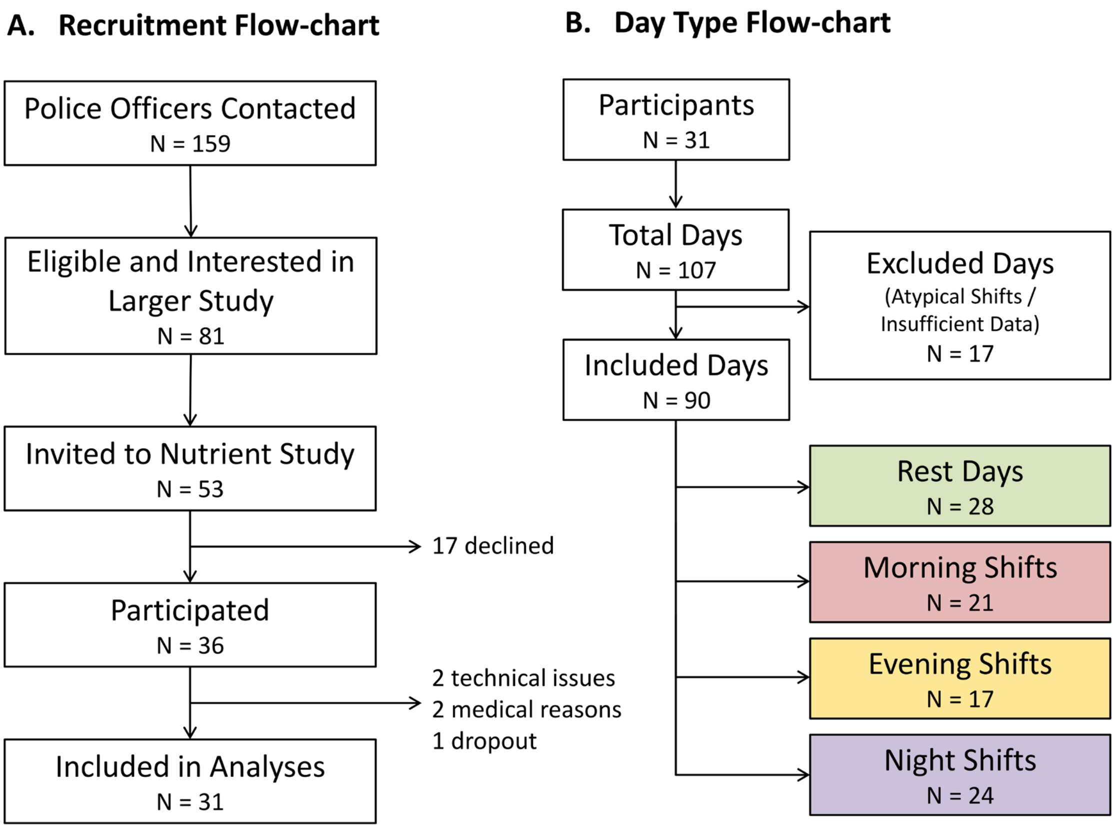


**Figure S1.** Flow-charts of (**A**) participant recruitment and (**B**) day types included in the final analyses. Days without a work period were classified as rest days. Work periods commencing between 0500 h and 0900 h were classified as morning shifts; those ending between 2200 h and 0200 h were classified as evening shifts; and those that encompassed the period between 0100 h and 0500 h were classified as night shifts. Atypical work periods longer than 13.5 h (i.e., overtime of more than half a 9-h shift) or shorter than 4.5 h (i.e., less than half a 9-h shift) were excluded from analyses.

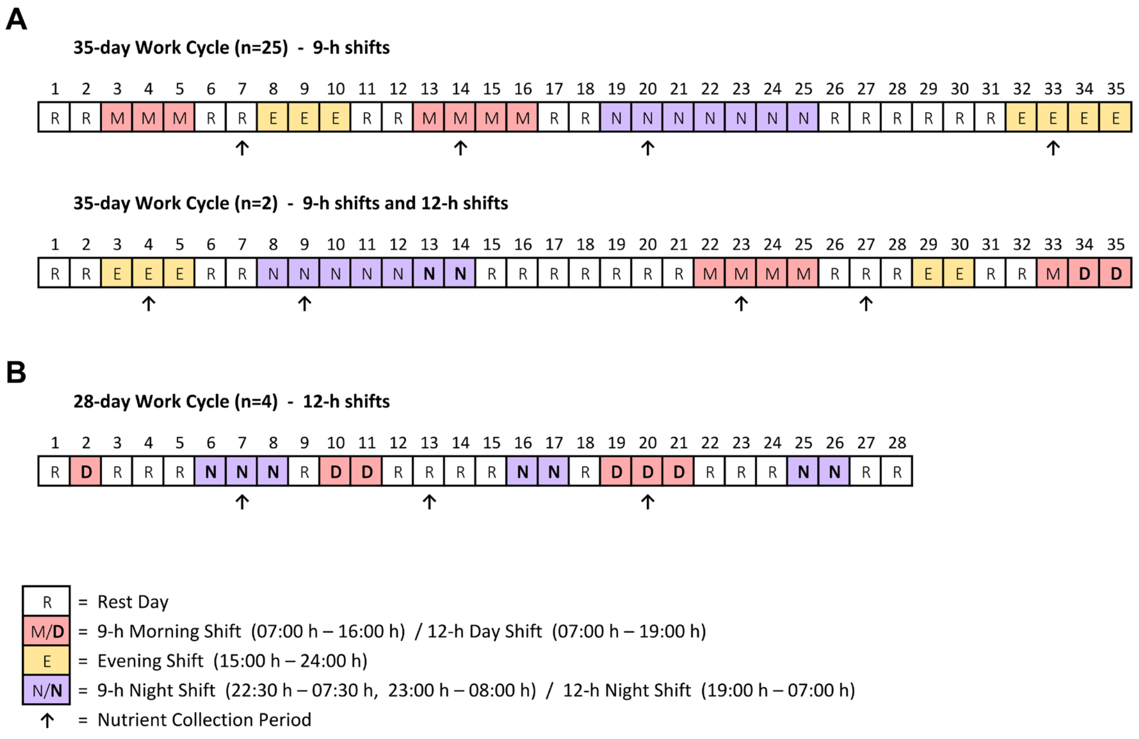


**Figure S2.** Diagram of participants' 35- and 28-day work rosters. (**A**) The 35-day roster (n=27) was composed of 9-h morning, evening, and night shifts. For 2 participants, the roster additionally comprised 12-h day and night shifts. (**B**) The 28-day roster (n=4) was composed of 12-h day and night shifts. Nutrient collection periods are indicated by arrows and were scheduled to occur on the second day in a sequence of shifts or rest days.

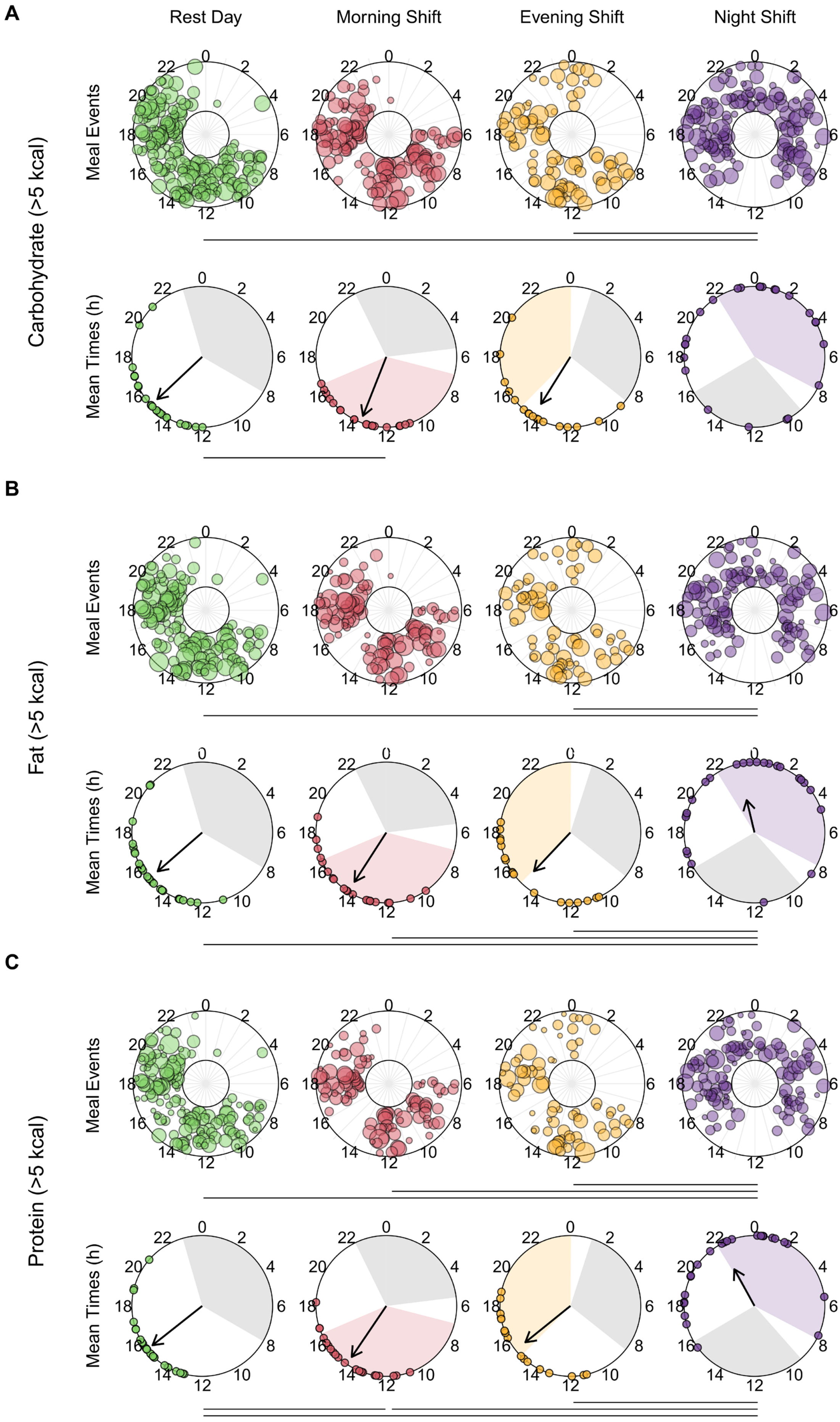
A
B
C
Rest Day
Morning Shift
Evening Shift
Night Shift
Carbohydrate (>5 kcal)
Fat (>5 kcal)
Protein (>5 kcal)
Meal Events
Mean Times (h)
0
2
4
6
8
10
12
14
16
18
20
22

**Figure S3.** Macronutrient events and mean times of macronutrient intake. (**A**) *Upper panel*: Meal events comprising >5 kcal of carbohydrate. *Lower panel*: Circular weighted mean times of carbohydrate intake. (**B**) *Upper panel*: Meal events comprising >5 kcal of fat. *Lower panel*: Circular weighted mean times of fat intake. (**C**) *Upper panel*: Meal events comprising >5 kcal of protein. *Lower panel*: Circular weighted mean times of protein intake.

*Upper Panels:* Individual macronutrient events (>5 kcal) are depicted as colored circles in concentric rings for each participant. The sizes of the colored circles reflect the macronutrient content of the meals. Horizontal lines represent significant (adjusted, $p<0.05$) differences in the spread or dispersion of macronutrient intake around the clock between day types. The spread per participant is derived from *Rho,* the mean resultant vector length of circular data. Protein intake was also more dispersed across night-shift days than morning-shift days (Table S3). Carbohydrate, fat, and protein intake were more dispersed across night-shift days than rest days and evening-shift days.

*Lower Panels:* Colored circles represent the circular mean time of macronutrient intake for each participant, weighted for the number of calories. Colored regions depict the average work hours for each shift type. The grey regions depict the average main sleep periods (based on wake and sleep times before and after each day, respectively). The directions of the arrows identify the group circular mean intake time for each macronutrient and day type. The lengths of the arrows reflect the spread of participants' mean intake times, with smaller arrows indicating a wider spread. No arrow is shown for carbohydrate intake because participant mean times were not non-uniformly distributed ($p=0.097$, Rayleigh test). Horizontal lines represent significant (adjusted, $p<.05$) differences in mean times between day types. On morning-shift days, the mean intake of carbohydrate and protein occurred earlier than on rest days (Table S3). On night-shift days, the mean intake of fat and protein occurred significantly later than on other days.

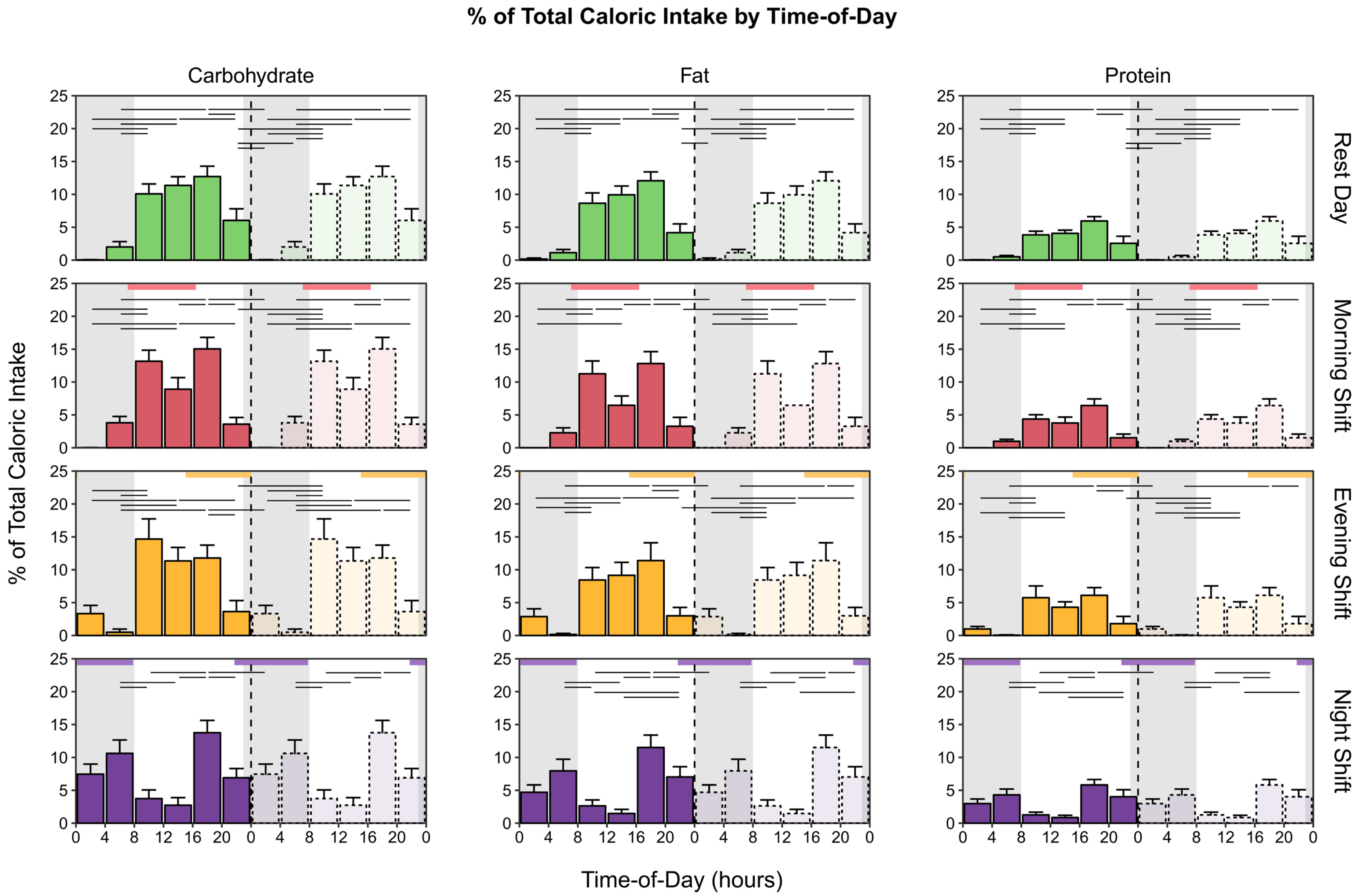


**Figure S4.** Distribution of macronutrient caloric intake by time-of-day for each day type. The percentages of total caloric intake from carbohydrates (*left panels*), fats (*middle panels*), and proteins (*right panels*) are double-plotted across the abscissa and illustrated by time-of-day (4-h bins). Grey regions represent the average sleep periods on rest days. Colored horizontal rectangles indicate the average work start and work end times for each shift type. Horizontal black lines indicate significant (adjusted, $p<0.05$) differences in the proportion of macronutrients consumed between time-of-day bins. Error bars represent standard error of the mean.

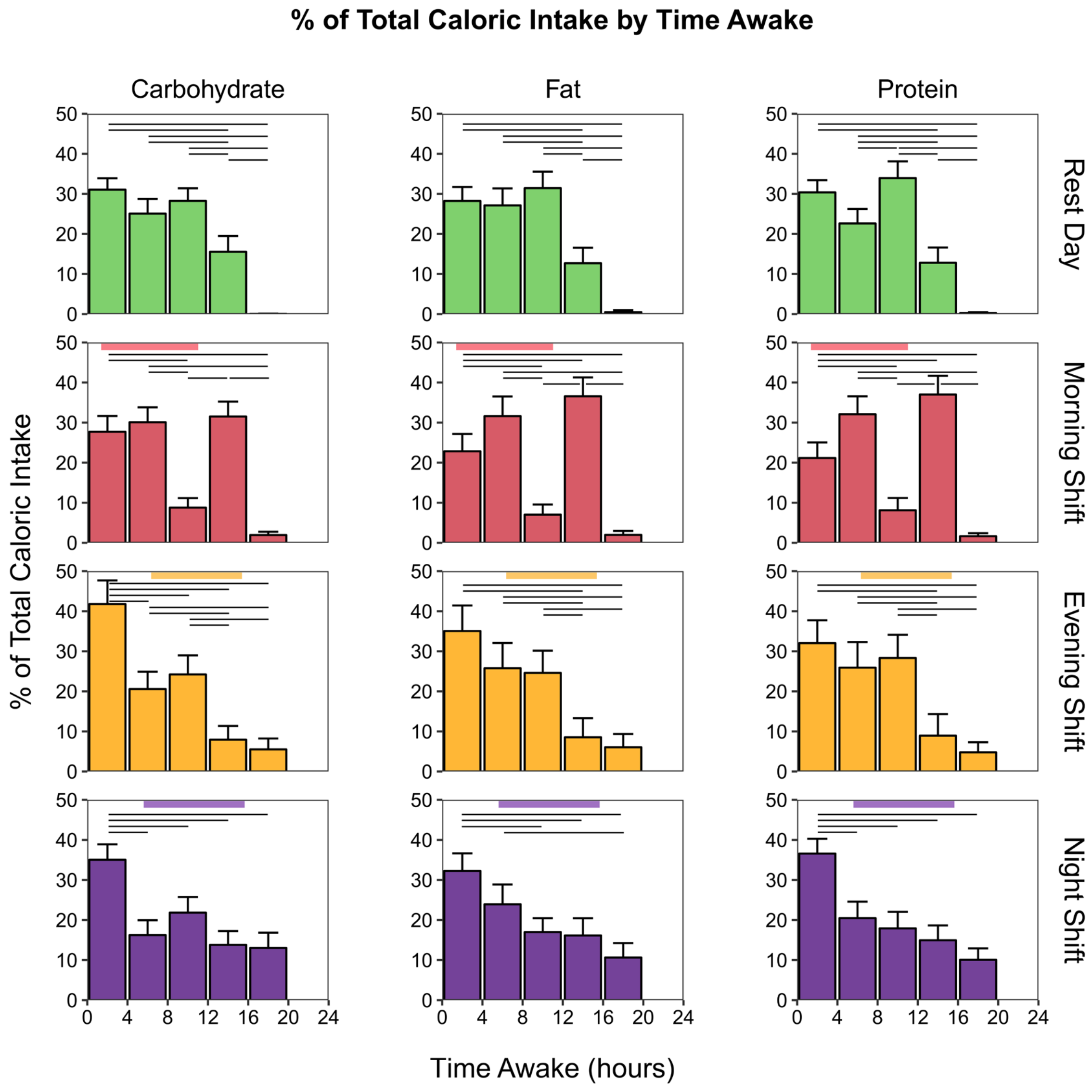


**Figure S5.** Distribution of macronutrient caloric intake by time awake for each day type. The percentages of total caloric intake from carbohydrates (left panels), fats (middle panels), and proteins (right panels) are plotted across the abscissa by time since waking from the prior main sleep period, adjusted for naps (4-h bins). Colored horizontal rectangles indicate the average work start and work end times for each shift type. Horizontal black lines indicate significant (adjusted, $p<0.05$) differences in the proportion of macronutrients consumed between times since waking bins. Error bars represent the standard error of the mean.
